# Current clinical practice in antibiotic treatment of *Staphylococcus aureus* bacteraemia: results from a survey in five European countries

**DOI:** 10.1093/jac/dkac237

**Published:** 2022-07-23

**Authors:** D T P Buis, J M Prins, L Betica-Radic, M G J de Boer, M Ekkelenkamp, D Kofteridis, N Peiffer-Smadja, J Schouten, N Spernovasilis, P Tattevin, J ten Oever, K C E Sigaloff

**Affiliations:** Amsterdam UMC, Vrije Universiteit Amsterdam, Department of Internal Medicine, Division of Infectious Diseases, Amsterdam Institute for Infection and Immunity, De Boelelaan 1117, Amsterdam, The Netherlands; Amsterdam UMC, Vrije Universiteit Amsterdam, Department of Internal Medicine, Division of Infectious Diseases, Amsterdam Institute for Infection and Immunity, De Boelelaan 1117, Amsterdam, The Netherlands; General Hospital Dubrovnik, Department of Infectious Diseases, University of Dubrovnik, Dubrovnik, Croatia; Leiden University Medical Center, Department of Infectious Diseases, Leiden, The Netherlands; UMC Utrecht, Department of Medical Microbiology, Heidelberglaan 100, 3584 CX Utrecht, The Netherlands; Department of Internal Medicine & Infectious Diseases, University Hospital of Heraklion, Heraklion, Greece; Infectious Disease Department, Bichat-Claude Bernard Hospital, Assistance-Publique Hôpitaux de Paris, Paris, France; Department of Intensive Care Medicine, Radboudumc, Nijmegen, The Netherlands; Department of Internal Medicine & Infectious Diseases, University Hospital of Heraklion, Heraklion, Greece; Department of Infectious Diseases, German Oncology Center, Limassol, Cyprus; Infectious Diseases and Intensive Care Unit, Pontchaillou University Hospital, Rennes, France; Radboud University Medical Center, Department of Internal Medicine and Radboud Center for Infectious Diseases, Geert Grooteplein Zuid 10, Nijmegen, The Netherlands; Amsterdam UMC, Vrije Universiteit Amsterdam, Department of Internal Medicine, Division of Infectious Diseases, Amsterdam Institute for Infection and Immunity, De Boelelaan 1117, Amsterdam, The Netherlands

## Abstract

**Objectives:**

To determine clinical practice variation and identify knowledge gaps in antibiotic treatment of *Staphylococcus aureus* bacteraemia (SAB).

**Methods:**

A web-based survey with questions addressing antibiotic treatment of SAB was distributed through the ESGAP network among infectious disease specialists, clinical microbiologists and internists in Croatia, France, Greece, the Netherlands and the UK between July 2021 and November 2021.

**Results:**

A total number of 1687 respondents opened the survey link, of whom 677 (40%) answered at least one question. For MSSA and MRSA bacteraemia, 98% and 94% preferred initial monotherapy, respectively. In patients with SAB and non-removable infected prosthetic material, between 80% and 90% would use rifampicin as part of the treatment. For bone and joint infections, 65%–77% of respondents would consider oral step-down therapy, but for endovascular infections only 12%–32% would. Respondents recommended widely varying treatment durations for SAB with different foci of infection. Overall, 48% stated they used ^18^F-fluorodeoxyglucose positron emission tomography/CT (18F-FDG-PET/CT) to guide antibiotic treatment duration. Persistent bacteraemia was the only risk factor for complicated SAB that would prompt a majority to extend treatment from 2 to 4–6 weeks.

**Conclusions:**

This survey in five European countries shows considerable clinical practice variation between and within countries in the antibiotic management of SAB, in particular regarding oral step-down therapy, choice of oral antibiotic agents, treatment duration and use of 18F-FDG-PET/CT. Physicians use varying criteria for treatment decisions, as evidence from clinical trials is often lacking. These areas of practice variation could be used to prioritize future studies for further improvement of SAB care.

## Introduction

*Staphylococcus aureus* bacteraemia (SAB) is an exceptionally heterogeneous disease and its clinical course can range from mild to life-threatening.^1^ This clinical heterogeneity is the result of complex interplay between pathogen and host factors, and is reflected by varying distributions of clinical phenotypes and mortality rates between observational cohort studies.^1,2^

In recent decades, evidence-based interventions including infectious disease (ID) consultation, follow-up blood cultures, echocardiography and source control interventions have improved clinical outcomes in SAB patients.^3–5^ Conversely, appropriate antibiotic treatment, one of the key components of SAB management, is still guided by evidence of poor quality.^1^ This is reflected in current guidelines on SAB, which vary widely in their recommendations regarding choice of antibiotic agents, treatment duration and optimal administration route.^6–9^

It is likely that the paucity of high-level evidence results in clinical practice variation in antibiotic treatment of SAB. In order to substantiate the clinical practice variation, a web-based survey was designed and distributed among ID specialists, clinical microbiologists and internists in five European countries. Areas of variation in management may indicate knowledge gaps, and identifying them may therefore guide future research efforts.

## Methods

### Survey development

We designed a web-based survey in two subsequent steps. First, we organized an online discussion with ID specialists and clinical microbiologists involved in SAB management to identify topics for inclusion in the survey. We developed a first draft of the survey with questions based on these topics. Second, this draft was tested in an independent group of ID specialists and clinical microbiologists. Their comments were used to adjust the survey and develop a final version (Appendix A, available as Supplementary data at *JAC* Online). The final version included 17 questions addressing the following topics: choice of antibiotic agents; use of rifampicin; oral antibiotic step-down therapy; and treatment duration. We also included two scenarios describing patients with SAB, with questions on recommended antibiotic treatment duration to illustrate clinical practice.

### Survey distribution

Target respondents included ID specialists, clinical microbiologists, internists and residents in training for one of these specialties. Five European countries were invited through the ESCMID Study Group for Antimicrobial Stewardship (ESGAP) to participate in the survey: the Netherlands, France, the UK, Greece and Croatia. These countries were chosen based on their geographical spread in Europe. We did not perform a formal sample size calculation, but aimed for at least 50 respondents per country. Distribution of the survey was initiated on 6 July 2021 and data collection was stopped on 30 November 2021. There were no financial or other incentives provided to the target respondents.

In all countries, the survey was shared by a web link and respondents were invited to forward the survey link to their colleagues. In the Netherlands, the survey was distributed through the Dutch Working Party on Antibiotic Policy, the Dutch Society for Infectious Disease specialists and the Dutch Society for Medical Microbiology. In France, the survey was shared via the French Society for Infectious Diseases, the French National Society for Internal Medicine, the French Young Infectious Diseases Network and the Young Internal Medicine Specialists Network. The survey was distributed in Croatia through the Croatian Society of Infectious Disease and the Croatian Society of Clinical Microbiology. In Greece, the survey was distributed through a network of ID specialists within the Greek Society for Infectious Diseases. In the UK, the survey was shared through the British Infection Association.

### Data analysis

A descriptive analysis of all survey questions was performed. Both complete and partially completed surveys were included in the data analysis. Continuous and categorical variables were presented as median (IQR) and absolute number (percentage), respectively. A sensitivity analysis was performed, excluding consultants in training. All analyses were performed using R version 4.0.3.

## Results

### Characteristics of respondents

The survey link was opened 1687 times. A total of 677 (40%) respondents answered at least one question concerning antibiotic treatment of SAB. Table 1 describes the demographic characteristics of these 677 respondents. Overall, 78% (527/677) of respondents completed the survey. Participants practised in France (45%), the Netherlands (18%), Greece (14%), the UK (13%), Croatia (5%) or another country (4%). Most respondents were ID specialists (56%), clinical microbiologists (21%) or internists (19%). Overall, 78% of respondents were registered consultants, while 22% were still in training. The majority of consultants had been registered for 0–10 years. Most respondents (65%) indicated they were involved in clinical decision-making in 11–50 cases of SAB per year and 18% were involved in more than 50 cases of SAB each year.

**Table 1.** Characteristics of survey respondents who answered at least one survey question (*n *= 677)

Characteristic	*n* (%)
Country	
France	306 (45)
The Netherlands	124 (18)
Greece	98 (14)
UK	86 (13)
Croatia	37 (5)
Other	26 (4)
Medical specialty	
ID	380 (56)
Clinical microbiology	143 (21)
Internal medicine	132 (19)
Other	22 (3)
Years registered as consultant	
In training for consultant	148 (22)
0–10	317 (47)
11–20	126 (19)
21–30	56 (8)
>30	30 (4)
Number of SAB cases respondent is involved in per year	
0–10	117 (17)
11–20	190 (28)
21–50	250 (37)
>50	120 (18)

### Antibiotic agents for MSSA bacteraemia

For initial treatment of monobacterial MSSA bacteraemia, 98% (664/677) favoured antibiotic monotherapy and 2% (13/677) combination therapy. Of those who preferred antibiotic monotherapy, 81% (536/664) indicated an anti-staphylococcal penicillin as their first-choice antibiotic agent and 16% (108/664) a first-generation cephalosporin (Figure S1). When treating a patient with MSSA bacteraemia without CNS infection, 74% (496/668) would consider first-generation cephalosporins to have equivalent clinical effectiveness as anti-staphylococcal penicillins. This percentage varied between 39% (33/84) and 91% (112/123) in the different countries (Table S1).

### Antibiotic agents for MRSA bacteraemia

When treating a patient with confirmed monobacterial MRSA bacteraemia, 94% (635/677) would prefer initial antibiotic monotherapy. Of these respondents, 71% (452/635) preferred a glycopeptide (e.g. vancomycin) as initial antibiotic therapy and 23% (148/635) a lipopeptide (e.g. daptomycin) (Figure S2). Respondents using combination therapy recommended widely varying regimens. In this group, the combination of a glycopeptide plus an aminoglycoside was the most used regimen (7/42; 17%).

### Use of rifampicin

In patients with SAB and non-removable infected prosthetic material, between 80% (448/560) and 90% (503/560) of respondents would use rifampicin as part of the treatment, provided the isolates were susceptible (Figure S3). For SAB and an associated infected joint prosthesis, 90% (503/560) would treat with a rifampicin-based therapy.

### Oral step-down therapy

**Figure 1.**
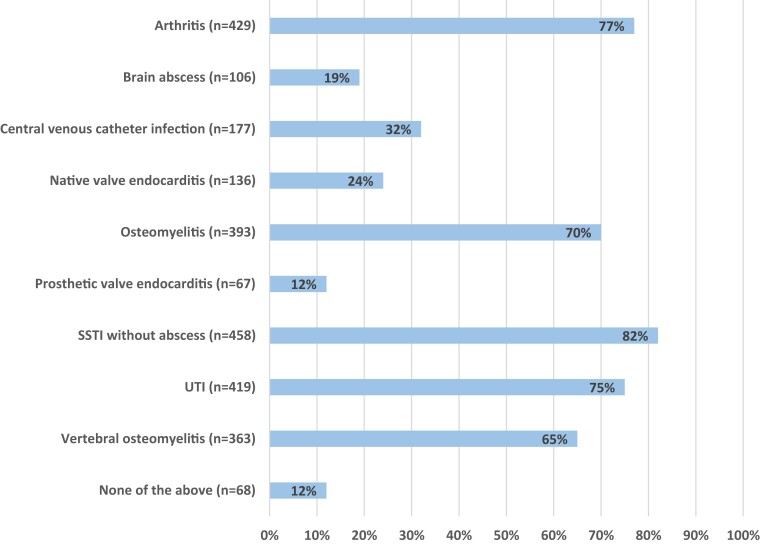
Foci of infection for which participants would consider oral step-down antibiotic therapy (*n *= 560). SSTI, skin-and-soft-tissue infection; UTI, urinary tract infection; *n*, the number of respondents who chose a specific answer option. This figure appears in colour in the online version of *JAC* and in black and white in the print version of *JAC*.

Figure 1 shows the percentage of respondents who would consider oral step-down antibiotic therapy per infectious focus. Only 12% (68/560) would not consider oral step-down antibiotic therapy in any of the infectious foci surveyed. For bone and joint infections, 65%–77% of respondents, and for skin-and-soft-tissue infections 82% (458/560) of respondents, would consider using oral step-down therapy. In patients with endovascular infections, including central vascular catheter infection and native and prosthetic valve endocarditis, 32% (177/560), 24% (136/560) and 12% (67/560) of respondents, respectively, would consider oral step-down antibiotics.

Most respondents considered clearance of bacteraemia 48–72 h after initiation of adequate antibiotic treatment (370/490; 76%) and absence of CNS infection (336/490; 69%) as requirements for oral step-down therapy, provided the patient is able to take oral medication (Figure S4). Transoesophageal echocardiography (TEE) without signs of endocarditis was more frequently reported as a requirement than normal transthoracic echocardiography (TTE) (252/490; 51% versus 197/490; 40%). Only a minority (116/490; 24%) would use initiation of adequate antibiotic therapy within 48 h after blood culture collection and ^18^F-fluorodeoxyglucose positron emission tomography/CT (18F-FDG-PET/CT) without signs of endocarditis or metastatic infections (120/490; 24%) as a requirement for using oral step-down therapy.

### Antibiotic agents for oral step-down therapy

When treating a patient with MSSA bacteraemia with oral step-down therapy, 65% (319/489) preferred monotherapy and 35% (170/489) combination therapy, assuming there is no implanted prosthetic material and the isolate is susceptible to the drug. Preferred agents for oral step-down were anti-staphylococcal penicillins and clindamycin (both 32%). Eleven percent (36/319) of respondents most commonly prescribed a fluoroquinolone and 10% (33/319) trimethoprim/sulfamethoxazole (Figure S5). For oral-step down therapy in a patient with MRSA bacteraemia, 68% (330/486) would recommend monotherapy over combination therapy. Linezolid (114/330; 35%), clindamycin (107/330; 32%) and trimethoprim/sulfamethoxazole (65/330; 20%) were most commonly prescribed as monotherapy (Figure S6). A fluoroquinolone plus rifampicin was the most used oral step-down combination regimen in both MSSA (39/170; 23%) and MRSA (39/156; 25%) bacteraemia.

### Antibiotic treatment duration

Table 2 shows respondents’ preferences concerning antibiotic treatment duration in patients with SAB without implanted prosthetic material. For arthritis, 17% (91/536) favoured 2 weeks, 48% (259/536) 4 weeks and 34% (182/536) 6 weeks of antibiotic treatment. When managing a patient with native valve endocarditis, 59% (317/536) indicated they would prefer 6 weeks of treatment and 37% (198/536) chose 4 weeks of treatment. The majority of respondents (448/536; 84%) would treat a patient with pneumonia without abscess for 2 weeks.

**Table 2.** Preferred antibiotic treatment duration in SAB patients without implanted prosthetic material (*n *= 536)

Antibiotic treatment duration (weeks)	Arthritis *n* (%)	Native valve endocarditis *n* (%)	Osteomyelitis *n* (%)	Pneumonia without abscess *n* (%)	Septic thrombophlebitis *n* (%)	Vertebral osteomyelitis without abscess *n* (%)
2	91 (17)	6 (1)	7 (1)	448 (84)	188 (35)	8 (1)
4	259 (48)	198 (37)	64 (12)	63 (12)	236 (44)	52 (10)
6	182 (34)	317 (59)	407 (76)	24 (4)	105 (20)	423 (79)
>6	4 (1)	15 (3)	58 (11)	1 (0)	7 (1)	53 (10)

Approximately half of respondents (255/536; 48%) stated they used 18F-FDG-PET/CT to guide antibiotic treatment duration in SAB. Respondents from Greece reported the lowest use of 18F-FDG-PET/CT (13/83; 16%) and respondents from the Netherlands the highest (97/106; 90%) (Table S2). Respondents were further asked to indicate which factors would make them consider extending antibiotic therapy from 2 to 4–6 weeks, assuming TEE did not show signs of endocarditis and, if they used it, 18F-FDG-PET/CT did not show signs of metastatic infection. Figure S7 displays the results for respondents using 18F-FDG-PET/CT and not using 18F-FDG-PET/CT separately. In both groups, a large majority [215/255, (84%) and 224/281 (80%), respectively] stated that persistent bacteraemia after 72 h of adequate treatment was a reason to extend treatment. For all other factors, only a minority of respondents stated it would make them consider extending antibiotic therapy. Respondents using 18F-FDG-PET/CT more frequently reported community acquisition of infection as a reason to extend treatment than respondents not using 18F-FDG-PET/CT [56/255 (22%) versus 34/281 (12%)].

### Scenarios

**Figure 2.**
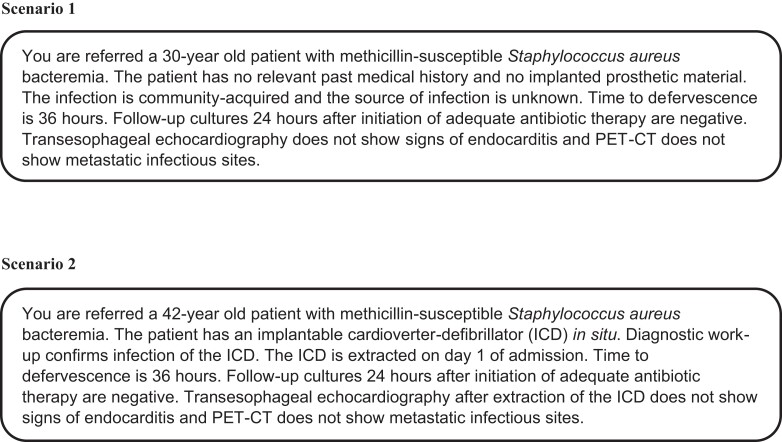
Scenarios describing patients with SAB.

Respondents were asked to provide a recommendation on duration of antibiotic treatment in two scenarios describing patients with SAB. A full description of both scenarios is provided in Figure 2. Overall, 527 respondents provided their recommend treatment durations. For scenario 1, recommended duration was 2 weeks for 89% (467/527) of respondents and 4 weeks for 8% (40/527) of respondents. For scenario 2, recommended durations of therapy were 2 weeks for 55% (292/527), 4 weeks for 23% (119/527) and 6 weeks for 18% (97/527) of respondents.

### Sensitivity analysis

No substantial differences were observed between the results of all respondents and those of registered consultants only, excluding those in training (data not shown).

## Discussion

This survey in five European countries showed considerable clinical practice variation between and within countries in the antibiotic management of SAB. Areas of consensus were choice of IV antibiotic agents, use of combination therapy and use of rifampicin-based treatment in prosthetic material infection. Main areas of controversy were eligibility for oral step-down therapy, choice of oral antibiotic agents, treatment duration and use of 18F-FDG-PET/CT.

### Antibiotic treatment of MSSA bacteraemia

A large majority of respondents preferred monotherapy for treatment of microbiologically confirmed MSSA and MRSA bacteraemia. Most respondents would use an anti-staphylococcal penicillin for MSSA bacteraemia. On the other hand, three-quarters of respondents considered first-generation cephalosporins to have equivalent clinical effectiveness to anti-staphylococcal penicillins in MSSA bacteraemia without CNS infection. This is considerably higher than in a previous study among ID specialists in the USA and Canada, of whom only 32% considered nafcillin and cefazolin equivalent in the treatment of left-sided MSSA endocarditis without CNS involvement.^10^ This difference might be explained by the recent publication of observational studies indicating comparable effectiveness of both agents and less antibiotic discontinuation due to adverse events in patients using cefazolin.^11,12^ Randomized clinical trials might provide stronger evidence about the equivalence of first-generation cephalosporins compared with anti-staphylococcal penicillins in SAB treatment.^13,14^

### Use of rifampicin

There was substantial agreement between respondents concerning the use of rifampicin for different types of prosthetic material infection. International guidelines recommend rifampicin as part of combination therapy for SAB and associated infected prosthetic material, including prosthetic valve endocarditis and prosthetic joint infections.^6,15^ The underlying evidence to support these recommendations is, however, limited since previous studies were either small or potentially suffered from confounding by indication.^16–18^ In the ARREST trial, adjunctive rifampicin provided no overall benefit over standard antibiotic therapy in SAB.^19^ However, only 14 (2%) patients in this study had infected prosthetic material, limiting the generalizability of the results to this subgroup. The consensus regarding use of rifampicin, despite a lack of high-quality studies, illustrates that agreement between respondents is not always a reflection of a solid evidence base. Since rifampicin-based therapies carry a high risk of adverse drug events and drug interactions, future studies demonstrating a robust effect of rifampicin on patient outcomes are needed to justify their ubiquitous use in clinical practice.

### Oral step-down therapy

A large majority indicated they would consider oral step-down antibiotic therapy in SAB. There was relative consensus that endovascular infections, including infective endocarditis and central venous catheter infection, should not be treated with oral antibiotics. On the other hand, a majority would consider oral step-down for non-endovascular infections including arthritis, osteomyelitis and skin-and-soft-tissue infection without abscess. This survey also highlights the fact that clinicians apply different criteria to selected patients who can be treated with oral antibiotics. Absence of CNS infection and absence of persistent bacteraemia were consistent requirements for oral step-down therapy, but there was substantial disagreement on other criteria. This may originate from the fact that there is limited evidence to support the use of oral antibiotics in SAB.^20^ Previous studies were performed in heterogeneous patient populations, included only small numbers of patients with SAB, or resulted in conflicting conclusions.^21–24^ For example, the POET trial on partial oral treatment of endocarditis included only 47 participants with *S. aureus* endocarditis who received oral treatment.^24^ We did not assess in our survey after how many days of IV therapy respondents would consider oral step-down, which might contribute to variation in respondents’ answers. Currently, several studies examine the efficacy and safety of oral switch therapy in SAB, including the SABATO trial and the SNAP trial (ClinicalTrials.gov NCT05137119), and the results are eagerly awaited to inform clinical practice.^25^

### Antibiotic treatment duration

Respondents recommended widely varying treatment durations for SAB with different foci of infection. A patient with septic arthritis and accompanying SAB would receive 4 weeks of treatment from approximately half of the respondents, 2 weeks from a fifth of respondents and 6 weeks from a third of respondents. The lack of consensus is also illustrated by respondents’ answers on the factors that influence their decision to extend antibiotic therapy from 2 to 4–6 weeks. Only persistent bacteraemia would prompt a majority to extend treatment. Even factors that previous studies identified as conferring a high risk for complications, such as persistent fever and delay in start of adequate antibiotic treatment, were mentioned by only a minority of respondents.^26^ A previous survey in the USA observed similar responses regarding the factors influencing extension of therapy.^10^ This lack of consensus may be caused by scarcity of high-quality studies, and is in line with a previous survey among ID physicians in Australia and New Zealand, which identified optimal duration of therapy as the number one research priority in SAB.^1,27^ Currently, randomized controlled trials are investigating whether antibiotic therapy can be safely shortened from 6 to 4 weeks in selected patients with complicated SAB (Netherlands Trial Register NL8347) and from 14 to 7 days in patients with uncomplicated SAB.^28^

### 18F-FDG-PET/CT

Recent studies suggest that performing 18F-FDG-PET/CT might be beneficial by detecting metastatic infections, with subsequent treatment modifications.^29,30^ Half of respondents in the survey indicated they used 18F-FDG-PET/CT in SAB management, with substantial differences between countries. These local differences might be explained by differences in availability of scanners and associated costs.^31,32^ Previous surveys in the USA and Australia and New Zealand did not include questions on use of 18F-FDG-PET/CT in SAB.^10,27^ Moreover, this survey provides insight into the way clinicians incorporate 18F-FDG-PET/CT results in decision-making in SAB management. Interestingly, we observed no major differences between respondents who used 18F-FDG-PET/CT and the group that did not, with the assumption of a negative 18F-FDG-PET/CT. These findings suggest that absence of metastatic infections on an 18F-FDG-PET/CT has at present very limited influence on the decision whether or not to extend treatment.

### Strengths and limitations

Strengths of this study are that we tested the survey format in an independent group of clinicians in the development phase, and obtained data from a large number of respondents from different geographical regions in Europe. We acknowledge that this study has limitations. First, since participation was voluntary, selection bias may limit generalizability. Second, clinical practices were self-reported and might not adequately reflect everyday real-life practice. Furthermore, we did not assess whether respondents were primarily involved in SAB care for adults, children or both. Since clinical practice might vary per patient age group, this might have biased the results. Last, almost half of the respondents practised in France and therefore the results might disproportionally reflect clinical practice in this country. To adjust for this, we performed exploratory analyses stratified per country (Tables S1 and S2).

### Conclusions

**Figure 3.**
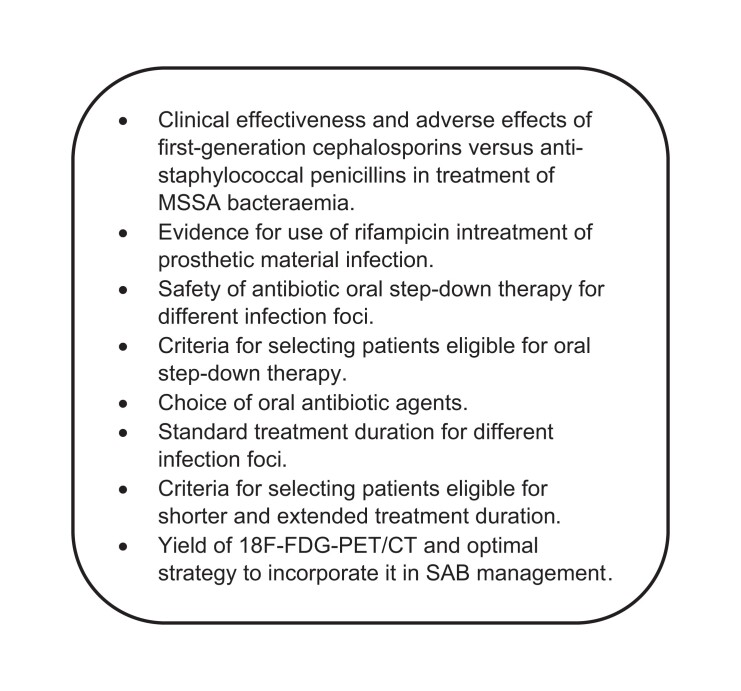
Main areas of controversy in antibiotic management of SAB.

This survey shows that considerable practice variation is present in Europe in antibiotic treatment of SAB. Clinicians have the difficult task of identifying patients who are eligible for certain targeted interventions, such as shorter, extended or oral step-down therapy. Physicians use varying criteria, as evidence from the literature is often lacking and virtually no guideline recommendations on SAB exist, which leads to clinical practice variation. This calls for methodologically sound and preferably randomized studies investigating interventions in well-defined subgroups of SAB patients. The difficulty of performing these studies is illustrated by the SABATO trial, for which enrolment started in 2013 and which was only completed in 2020 after decreasing the target sample size.^25^ Nevertheless, such studies are much needed for guidance of clinical decision-making. The final step is to implement existing and future evidence into clinical practice, as practice variation is not only determined by lack of evidence. This survey indicates the main areas of practice variation, which could be used to direct and prioritize future studies for further improvement of SAB care (Figure 3).

## Supplementary Material

dkac237_Supplementary_Data

## Supplementary data

Appendix A, Tables S1 and S2 and Figures S1 to S7 are available as Supplementary data at *JAC* Online.
