# Supplementary material for: Current clinical practice in antibiotic treatment of Staphylococcus aureus bacteraemia: results from a survey in five European countries
Source: J Antimicrob Chemother. 2022 Jul 23;77(10):2827–34. doi: 10.1093/jac/dkac237 (PMC9797040; doi:10.1093/jac/dkac237)
Supplement: dkac237_Supplementary_Data [file dkac237_supplementary_data.docx]

**Supplementary data**

**Appendix A: Complete survey**

1) In which country do you currently practice?

- Austria
- Belgium
- Bulgaria
- Croatia
- Republic of Cyprus
- Czech Republic
- Denmark
- Estonia
- Finland
- France
- Germany
- Greece
- Hungary
- Ireland
- Italy
- Latvia
- Lithuania
- Luxembourg
- Malta
- Netherlands
- Poland
- Portugal
- Romania
- Slovakia
- Slovenia
- Spain
- Sweden
- Other

2) Which country? (If question 1 = “Other”)

__________

3) Which of the following best describes your primary area of medical specialty?

- Clinical microbiology
- Infectious diseases
- Internal medicine
- Other

4) Please specify. (If question 3 = “Other”)

__________

5) How many years have you been registered as a consultant (i.e. medical specialist)?

- Still in training for consultant
- 0-10 years
- 11-20 years
- 21-30 years
- More than 30 years

6) Approximately in how many cases of *Staphylococcus aureus* bacteremia are you involved in clinical decision making per year?

- 0-10 cases
- 11-20 cases
- 21-50 cases
- More than 50 cases

7) Do you routinely use antibiotic monotherapy or antibiotic combination therapy in patients with confirmed monobacterial **MSSA bacteremia** without implanted prosthetic material?

- Antibiotic monotherapy
- Antibiotic combination therapy

8) What is your first-choice initial antibiotic regimen in patients with confirmed monobacterial **MSSA bacteremia** without implanted prosthetic material provided the isolate is **susceptible** to the drug? Mark all that apply. (If question 7 = “Antibiotic combination therapy”)

- Aminoglycoside, e.g. gentamicin
- Anti-staphylococcal penicillin, e.g. flucloxacillin
- Carbapenem, e.g. meropenem
- Clindamycin
- First-generation cephalosporin, e.g. cefazoline
- Second-generation cephalosporin, e.g. cefuroxime
- Third-generation cephalosporin, e.g. ceftriaxone
- Fourth-generation cephalosporin, e.g. cefepime
- Fifth generation cephalosporin, i.e. ceftaroline
- Fluoroquinolone, e.g. levofloxacin
- Glycopeptide, e.g. vancomycin
- Linezolid
- Lipopeptide, e.g. daptomycin
- Piperacillin/tazobactam
- Rifampicin
- Trimethoprim/sulfamethoxazole
- Other

9) Please specify. (If question 8 = “Other”)

__________

10) What is your first-choice initial antibiotic regimen in patients with confirmed monobacterial MSSA bacteremia without implanted prosthetic material provided the isolate is susceptible to the drug? (If question 7 = “Antibiotic monotherapy”)

- Anti-staphylococcal penicillin, e.g. flucloxacillin
- Carbapenem, e.g. meropenem
- Clindamycin
- First-generation cephalosporin, e.g. cefazoline
- Second-generation cephalosporin, e.g. cefuroxime
- Third-generation cephalosporin, e.g. ceftriaxone
- Fourth-generation cephalosporin, e.g. cefepime
- Fifth generation cephalosporin, i.e. ceftaroline
- Glycopeptide, e.g. vancomycin
- Lipopeptide, e.g. daptomycin
- Piperacillin/tazobactam
- Other

11) Please specify. (If question 10 = “Other”)

__________

12) Do you routinely use antibiotic monotherapy or antibiotic combination therapy in patients with confirmed monobacterial **MRSA bacteremia** without implanted prosthetic material?

- Antibiotic monotherapy
- Antibiotic combination therapy

13) What is your first-choice initial antibiotic regimen in patients with confirmed monobacterial **MRSA bacteremia** without implanted prosthetic material provided the isolate is **susceptible** to the drug? Mark all that apply. (If question 12 = “Antibiotic combination therapy”)

- Aminoglycoside, e.g. gentamicin
- Clindamycin
- Fifth generation cephalosporin, i.e. ceftaroline
- Fluoroquinolone, e.g. levofloxacin
- Fosfomycin
- Glycopeptide, e.g. vancomycin
- Linezolid
- Lipopeptide, e.g. daptomycin
- Rifampicin
- Tetracyclin, e.g. doxycycline
- Trimethoprim/sulfamethoxazole
- Other

14) Please specify. (If question 13 = “Other”)

__________

15) What is your first-choice initial antibiotic regimen in patients with confirmed monobacterial **MRSA bacteremia** without implanted prosthetic material provided the isolate is susceptible to the drug? (If question 12 = “Antibiotic monotherapy”)

- Fifth generation cephalosporin, i.e. ceftaroline
- Glycopeptide, e.g. vancomycin
- Linezolid
- Lipopeptide, e.g. daptomycin
- Trimethoprim/sulfamethoxazole
- Other

16) Please specify. (If question 15 = “Other”)

__________

17) Do you consider first-generation cephalosporins (e.g. cefazoline) to have equivalent clinical effectiveness for **MSSA bacteremia without central nervous system infection** as anti-staphylococcal penicillins ( e.g. flucloxacillin)?

- Yes
- No

18) Do you treat patients with *Staphylococcus aureus* bacteremia and **the following types of infected prosthetic material** which will not be removed with **rifampicin** as part of combination antibiotic therapy provided the isolate is susceptible to the drug? Mark all that apply.

- Cardiac device
- Endovascular graft
- Joint prosthesis
- Prosthetic heart valve
- Spondylodesis
- All of the above
- None of the above

19) Would you consider oral step-down antibiotic therapy in patients with *Staphylococcus aureus* bacteremia and the following foci of infection? Mark all that apply.

- Arthritis
- Brain abscess
- Central line infection
- Native valve endocarditis
- Osteomyelitis
- Prosthetic valve endocarditis
- Skin- and soft tissue infection without abscess
- Urinary tract infection
- Vertebral osteomyelitis
- None of the above

20) In your opinion, which of the following criteria must a patient with *Staphylococcus aureus* bacteremia who is able to take oral medication fulfill to be eligible for oral step-down antibiotic therapy? Mark all that apply. (If question 19 ≠ “None of the above”)

- Absence of central nervous system infection
- Absence of endovascular infection focus other than endocarditis
- Blood culture negativity 48-72 hours after initiation of adequate antibiotic treatment
- Defervescence within 72 hours
- Hospital acquired bacteremia
- Initiation of adequate antibiotic treatment within 48 hours of blood culture collection
- PET-CT without signs of endocarditis and metastatic infections
- Transesophageal echocardiography (TEE) without signs of endocarditis
- Transthoracal echocardiography (TTE) without signs of endocarditis
- None of the above

21) If oral drugs are acceptable in your opinion, what is your **most commonly** prescribed antibiotic regimen for oral step-down therapy in patients with confirmed **MSSA** bacteremia **without** implanted prosthetic material provided the isolate is **susceptible** to the drug? Choose only one answer, unless you routinely prescribe combination therapy. In that case mark all that apply. (If question 19 ≠ “None of the above”)

- Anti-staphylococcal penicillin, e.g. flucloxacillin
- Clindamycin
- Fluoroquinolone, e.g. levofloxacin
- Fusidic acid
- Linezolid
- Macrolide, e.g. erythromycin
- Penicillin, e.g. amoxicillin
- Rifampicin
- Tetracyclin, e.g. doxycycline
- Trimethoprim/sulfamethoxazole
- Other

22) Please specify. (If question 21 = “Other”

__________

23) If oral drugs are acceptable in your opinion, what is what is your most commonly prescribed antibiotic regimen for oral step-down therapy in patients with confirmed MRSA bacteremia without implanted prosthetic material provided the isolate is susceptible to the drug? Choose only one answer, unless you routinely prescribe combination therapy. In that case mark all that apply. (If question 19 ≠ “None of the above”)

- Clindamycin
- Fluoroquinolone, e.g. levofloxacin
- Fusidic acid
- Linezolid
- Macrolide, e.g. erythromycin
- Rifampicin
- Tetracyclin, e.g. doxycycline
- Trimethoprim/sulfamethoxazole
- Other

24) Please specify. (If question 23 = “Other”

__________

25) How many weeks of antibiotic treatment would you prescribe in a patient with *Staphylococcus aureus* bacteremia **without implanted prosthetic material** and the following foci of infection?

|  | 2 weeks | 4 weeks | 6 weeks | More than 6 weeks |
| --- | --- | --- | --- | --- |
| Arthritis |  |  |  |  |
| Native valve endocarditis |  |  |  |  |
| Osteomyelitis |  |  |  |  |
| Pneumonia without abscess |  |  |  |  |
| Septic thromboplebitis |  |  |  |  |
| Spondylodiscitis without abscess |  |  |  |  |

26) Do you ever use PET-CT to guide antibiotic treatment duration in patients with *Staphylococcus aureus* bacteremia?

- Yes
- No

27) Would the following factors make you consider extending antibiotic therapy from 2 weeks to 4-6 weeks in a patient with *Staphylococcus aureus* bacteremia? Assume transesophageal echocardiography (TEE) does not show signs of endocarditis. (If question 26= ”No”)

|  | Yes | No |
| --- | --- | --- |
| Community-acquisition |  |  |
| Delay of 48 hours between sampling first positive blood culture and initiation of adequate antibiotic treatment |  |  |
| Fever at 72 hours after first positive blood culture |  |  |
| Positive blood cultures after 72 hours of adequate antibiotic treatment |  |  |
| Unknown portal of entry |  |  |
| Methicillin resistant *Staphylococcus aureus* |  |  |
| Age > 75 years |  |  |
| Immunocompromised patient |  |  |

28) Would the following factors make you consider extending antibiotic therapy from 2 weeks to 4-6 weeks in a patient with *Staphylococcus aureus* bacteremia? Assume transesophageal echocardiography (TEE) does not show signs of endocarditis and PET-CT does not show signs of metastatic infection. (If question 27= ”Yes”)

|  | Yes | No |
| --- | --- | --- |
| Community-acquisition |  |  |
| Delay of 48 hours between sampling first positive blood culture and initiation of adequate antibiotic treatment |  |  |
| Fever at 72 hours after first positive blood culture |  |  |
| Positive blood cultures after 72 hours of adequate antibiotic treatment |  |  |
| Unknown portal of entry |  |  |
| Methicillin resistant *Staphylococcus aureus* |  |  |
| Age > 75 years |  |  |
| Immunocompromised patient |  |  |

**Scenarios**

The following questions present patient cases with *Staphylococcus aureus* bacteremia. Please provide your recommendations concerning the cases.

You are referred a 30-year old patient with methicillin susceptible Staphylococcus aureus bacteremia. The patient has no relevant past medical history and no implanted prosthetic material. The infection is community-acquired and the source of infection is unknown. Time to defervescence is 36 hours. Follow-up cultures 24 hours after initiation of adequate antibiotic therapy are negative. Transesophageal echocardiography does not show signs of endocarditis and PET-CT does not show metastatic infectious sites.

How many weeks of antibiotic treatment would you recommend for this patient?

__________

You are referred a 42-year old patient with methicillin susceptible Staphylococcus aureus bacteremia. The patient has an implantable cardioverter-defibrillator (ICD) in situ. Diagnostic work-up confirms infection of the ICD. The ICD is extracted on day 1 of admission. Time to defervescence is 36 hours. Follow-up cultures 24 hours after initiation of adequate antibiotic therapy are negative. Transesophageal echocardiography after extraction of the ICD does not show signs of endocarditis and PET-CT does not show metastatic infectious sites.

How many weeks of antibiotic treatment would you recommend for this patient?

__________

**Figure S1** Preferred initial monotherapy regimen for monobacterial MSSA bacteremia (n=664).

MSSA, Methicillin Susceptible *Staphylococcus aureus*.
n equals number of respondents who chose a specific answer option.

**Figure S2** Preferred initial monotherapy regimen for monobacterial MRSA bacteremia (n=635).

MRSA, Methicillin Resistant *Staphylococcus aureus*.
n equals number of respondents who chose a specific answer option.

**Figure S3** Use of rifampicin combination therapy for different types of infected prosthetic material (n=560).

n equals number of respondents who chose a specific answer option.

**Figure S4** Requirements for use of oral step-down therapy (n=490).

SAB, *Staphylococcus aureus* bacteremia; IE, infective endocarditis; BC, blood culture; h, hours; AB, antibiotic; PET-CT; [18F]FDG-PET/CT; TEE, transesophageal echocardiography; TTE, transthoracic echocardiography.
n equals number of respondents who chose a specific answer option.

**Figure S5** Most commonly prescribed monotherapy regimens for oral step-down in MSSA bacteremia* (n=319).

*Without implanted prosthetic material and provided the isolate is susceptible to the drug.
MSSA, Methicillin Susceptible *Staphylococcus aureus*.
n equals number of respondents who chose a specific answer option.

**Figure S6** Most commonly prescribed monotherapy regimens for oral step-down in MRSA bacteremia* (n=330).

*Without implanted prosthetic material and provided the isolate is susceptible to the drug.
MRSA, Methicillin Resistant *Staphylococcus aureus*.
n equals number of respondents who chose a specific answer option.

**Figure S7** Factors influencing decision to extend antibiotic therapy from 2 to 4-6 weeks* (n=536).

SAB, *Staphylococcus aureus* bacteremia; PET-CT; [18F]FDG-PET/CT; AB, antibiotic; h, hours; BC, blood culture.
Results are reported separately for respondents using PET-CT and not using PET-CT.
*Assuming TEE does not show sign of endocarditis and PET-CT (for the group that uses PET/CT in clinical practice) does not show signs of metastatic infection.

**Table S1** Equivalent clinical effectiveness of ASP’s vs first-generation cephalosporins in MSSA bacteremia, per country (n=643).

|  | **Croatia** | **France** | **Greece** | **Netherlands** |  | **United Kingdom** |
| --- | --- | --- | --- | --- | --- | --- |
| **Yes** | 22 (59%) | 264 (87%) | 47 (48%) | 112 (91%) |  | 33 (39%) |
| **No** | 15 (41%) | 38 (13%) | 50 (52%) | 11 (9%) |  | 51 (61%) |

ASP, anti-staphylococcal penicillins; MSSA, Methicillin Susceptible *Staphylococcus aureus*.

**Table S2** Use of [18F]FDG-PET/CT to guide antibiotic treatment duration in SAB, per country (n=516).

|  | **Croatia** | **France** | **Greece** | **Netherlands** | **United Kingdom** |
| --- | --- | --- | --- | --- | --- |
| **Yes** | 7 (22%) | 100 (45%) | 13 (16%) | 97 (90%) | 31 (44%) |
| **No** | 25 (78%) | 122 (55%) | 70 (84%) | 9 (10%) | 40 (56%) |

SAB, *Staphylococcus aureus* bacteremia.
